# Interoperability Among Unmanned Maritime Vehicles: Review and First In-field Experimentation

**DOI:** 10.3389/frobt.2020.00091

**Published:** 2020-07-14

**Authors:** Riccardo Costanzi, Davide Fenucci, Vincenzo Manzari, Michele Micheli, Luca Morlando, Daniele Terracciano, Andrea Caiti, Mirko Stifani, Alessandra Tesei

**Affiliations:** ^1^DII (Dipartimento di Ingegneria dell'Informazione), Università di Pisa, Pisa, Italy; ^2^Marine Autonomous & Robotic Systems, National Oceanography Centre (NOC), Southampton, United Kingdom; ^3^CSSN (Centro di Supporto e Sperimentazione Navale), Italian Navy, La Spezia, Italy; ^4^NATO STO CMRE (Science & Technology Organization—Centre for Maritime Research and Experimentation), La Spezia, Italy

**Keywords:** autonomous underwater vehicle, marine robotics, NATO experimentation, robotic middleware, unmanned vehicles interoperability, Unmanned Maritime Vehicles

## Abstract

Complex maritime missions, both above and below the surface, have traditionally been carried out by manned surface ships and submarines equipped with advanced sensor systems. Unmanned Maritime Vehicles (UMVs) are increasingly demonstrating their potential for improving existing naval capabilities due to their rapid deployability, easy scalability, and high reconfigurability, offering a reduction in both operational time and cost. In addition, they mitigate the risk to personnel by leaving the man far-from-the-risk but in-the-loop of decision making. In the long-term, a clear interoperability framework between unmanned systems, human operators, and legacy platforms will be crucial for effective joint operations planning and execution. However, the present multi-vendor multi-protocol solutions in multi-domain UMVs activities are hard to interoperate without common mission control interfaces and communication protocol schemes. Furthermore, the underwater domain presents significant challenges that cannot be satisfied with the solutions developed for terrestrial networks. In this paper, the interoperability topic is discussed blending a review of the technological growth from 2000 onwards with recent authors' in-field experience; finally, important research directions for the future are given. Within the broad framework of interoperability in general, the paper focuses on the aspect of interoperability among UMVs not neglecting the role of the human operator in the loop. The picture emerging from the review demonstrates that interoperability is currently receiving a high level of attention with a great and diverse deal of effort. Besides, the manuscript describes the experience from a sea trial exercise, where interoperability has been demonstrated by integrating heterogeneous autonomous UMVs into the NATO Centre for Maritime Research and Experimentation (CMRE) network, using different robotic middlewares and acoustic modem technologies to implement a multistatic active sonar system. A perspective for the interoperability in marine robotics missions emerges in the paper, through a discussion of current capabilities, in-field experience and future advanced technologies unique to UMVs. Nonetheless, their application spread is slowed down by the lack of human confidence. In fact, an interoperable system-of-systems of autonomous UMVs will require operators involved only at a supervisory level. As trust develops, endorsed by stable and mature interoperability, human monitoring will be diminished to exploit the tremendous potential of fully autonomous UMVs.

## 1. Introduction

Unmanned Maritime Vehicles (UMVs) technology is increasingly demonstrating its potential to enhance existing naval capabilities, relying heavily on aircraft, helicopters, surface ships, and submarines to perform complex tasks. The integration with easily deployable, scalable systems of multiple UMVs offers an improvement in operation time, reduction of cost and mitigation of risk to personnel by leaving the man far-from-the-risk but in-the-loop of decision making. The achievement of the full potential of unmanned and autonomous systems must take into account the necessity of multi-national, multi-domain operations with multi-vendor, multi-protocol systems. The design of a clear framework for the interoperability of systems, both among them and with human operators, is essential toward effective planning and success of joint operations. On the other hand, UMVs in complex operational experimentations are hard to operate as an organic system-of-systems due to the expansion of non-standard solutions for mission control interface of the UMVs. In addition, the underwater domain poses significant communication challenges, such as multipath arrival structure, channel spread, and low data exchange rates.

In this complex framework, the definition of *Interoperability* is a tricky task *per se*. In order to share a common understanding, the National Institute of Standards and Technology definition is taken as a benchmark (Huang, 2004); the *Interoperability* is the ability of software or hardware systems to operate together successfully with minimal effort by the end-users, and it can be categorized into levels, types, or degrees of interoperability. It is pointed out that full interoperability would be facilitated by common or standard interfaces that are missing nowadays. section 2 and references therein provide an overview of the state-of-the-art of interoperability among UMVs, not neglecting the role of the human operator in the loop within the specific maritime domain, setting up a fundamental background for the reader.

The interoperability issue was approached in the Anti-Submarine Warfare—Operational Deployment of Concepts 2017 (ASW-ODC17) sea trial exercise, conducted in October 2017 off the coast of La Spezia (Italy). The sea trials were organized in the context of the Centre for Maritime Research and Experimentation (CMRE) of the NATO project Maritime Unmanned Systems (MUS) for ASW, involving NATO Naval Units and the Italian SEALab consortium. The SEALab is a joint laboratory between the Naval Support and Experimentation Center of the Italian Navy and the Italian Interuniversity Center of Integrated Systems for the Marine Environment (Terracciano et al., 2019). The goal of the MUS project is the development and verification at sea of a heterogeneous autonomous ASW network based on UMVs implementing a multistatic active sonar system. From the Italian point of view, the goal was to demonstrate the interoperability of a national Autonomous Underwater Vehicle [AUV Folaga WAVE, Fenucci et al. (2016)] within the CMRE robotic network for ASW (LePage et al., 2015) during a NATO operational exercise with assets of different NATO Navies. In-depth descriptions of the experimentation, high-level systems architectures and related software, and the specific interoperability experimental results are given in section 3.

Section 4 discusses the future challenges of interoperability, defining the current critical problems in marine robotics. Section 5 draws conclusions about interoperability among UMVs, merging the research advancement made over the past 20 years with the expertise of the authors and relevant guidelines for the future.

## 2. Interoperability Background and Relevant Literature

Sensors, platforms, software, and vehicle technologies are rapidly evolving, as well as processing and algorithm development, often outpacing the operational community capability to apply these new concepts in the field. In order to provide a broad view of the state of the art of interoperability, summaries—to be deepened with the cited bibliographical references—are provided below for the following topics:

- Adaptive Autonomous Communications and Networking;
- Command and Control System (C2S) and UMVs system-of-systems;
- Verification, Validation and Accreditation (VV&A) along with Modeling and Simulation (M&S);
- Interoperability Standardization;
- Robotics Middlewares.

It is necessary to stress the fact that these topics are all interconnected: only a synergic development of all of them enables a high level of interoperability.

Nowadays, research on *acoustic networking* is very engaged in supporting cooperative multi-vehicle missions which are increasingly dependent on the vehicles ability to inter-communicate. This must be accomplished exploiting and fusing the well-characterized Radio-Frequency (RF) channel with the time and space varying acoustic one. Ensuring the correct reception of a low bandwidth underwater acoustic signal affected by heavy delays and multipath interference is very challenging and error-prone, and it may result in limited interoperability among UMVs (Stojanovic, 2006). Recently, Caiti et al. (2012) provided a remarkable illustration of a persistent acoustic communication network with heterogeneous platform and sensors, both fixed and mobile; Been et al. (2010) presented collaborative distributed ASW operations performed by a scalable and autonomous networking system from a comprehensive scientific and end-user point of view.

Communications are necessary to address the present knowledge representation that is still embryonic and is intended for basic single platform and single domain applications. This limits the possibility of multiple coordinated mission between UMVs, i.e., they essentially collect data from sensors. In addition, the data collected during the mission are then typically processed offline. However, greater autonomy requires distributed service-oriented agents that need access to higher data representation levels. To our knowledge, the work reported in Miguelañez et al. (2011) was the first example of online underwater mission adaption thanks to a goal-based planning using semantic representation. A semantic-based framework was presented in this paper, which provides the central architecture for the representation of information in embedded autonomous agents. A pool of hierarchical ontologies to represent the information derived from the sensor data is used in the proposed architecture. The key benefit is that service-oriented agents can have access to various types of knowledge and can also contribute to its advancement in an interoperable way. For example, if the required information is not accessible to an agent due to poor communication in case of unfavorable underwater acoustic channel, the architecture provides the facility to request that the information be produced by other agents with the appropriate capabilities. The framework was also validated and assessed in a Mine Counter Measure (MCM) scenario, where ontological information representation, model-based diagnostics and adaptive mission techniques are integrated.

From the point of view of reconfigurable and adaptive communication networks, it is important to recall the SUNRISE (Sensing, monitoring and actuating on the UNderwater world through a federated Research InfraStructure Extending the Future Internet, http://fp7-sunrise.eu) European project (Braga et al., 2016). The SUNRISE consortium has, in particular, established an abstraction layer which enables the interaction between networking and communication components and the control software of different UMVs. Any networking or control program may use this interface mechanism, defined as Software-to-Software Communication (SSC), and an XML document (eXtended Markup Language) is used to define command structure and semantics. This method aims to combine control software with underwater communication and networking elements such that underwater networks can be more dynamic, versatile and efficient. The SSC protocol was fully tested and assessed in lab for all the robotics middlewares mentioned in section 2. In 2014 and 2015, sea experiments have also been performed in Porto, the Atlantic Ocean and the Mediterranean Sea. The SUNRISE redeployable testing facility was robust, simple to use and highly adaptable to different requirements, and a network of up to eight heterogeneous nodes were deployed during those sea trials. The SUNRISE open architecture allows additional hardware [e.g., sensor(s), battery pack(s), modem(s), external disk(s)], requested by the mission, to be quickly fitted on every node of the testbed. SUNRISE was one of the biggest demonstrations of the capabilities provided by the forthcoming Underwater Software-Defined Open-Architecture Modem (SDOAM) framework. The current state of these developments mainly involves academic and industrial R&D prototypes, while most of the commercial modems currently available are not “open” for reconfiguration and user programming (Dol et al., 2014), i.e., their physical-layer algorithms are hardcoded in the modem firmware. Since 2011, the CMRE has been promoting the introduction of SDOAMs and recently published a study outlining SDOAM development and deployment activities, as well as future directions (Potter et al., 2014). Part of the CMRE activities was made in the SUNRISE project. The CMRE SDOAM concept includes a policy engine that handles several protocols for all layers of the OSI stack. This, in effect, was a starting point for the evolution of cognitive architectures. In fact, the CMRE communication stack is evolving in a fully cognitive communications architecture (CCA) that uses intelligent, adaptive and secure underwater networking techniques (Petroccia et al., 2018).

A further element in interoperability studies is *the design of C2S* to support autonomous collaborative tasks. In the area of adaptive control of heterogeneous UMVs to find suitable solutions for their interoperability, the Massachusetts Institute of Technology (MIT) research team has developed a uniform approach (the Generic Ocean Array Technology Sonar—GOATS program Bovio et al., 2001) to command all the assets through a hierarchical structure capable of ensuring the data propagation in the entire network (Benjamin et al., 2010; Schneider and Schmidt, 2010). Alongside the advances in underwater acoustics communications, both in-field and analytical works on UMVs swarms cooperation have made significant progress with several initiatives supported by the European Union, e.g., the “Cooperative Cognitive Control for Autonomous Underwater Vehicles” (Co3AUVs) project (Birk et al., 2011).

EU projects such as GREX (Kalwa, 2010) present this kind of approach for hydrographic mapping where the vehicle surveys the seabed or water column utilizing sonars and other sensors. However, there are certain shortcomings in such unmanned surveys, including the rate of acquisition and the limited swath width of the high-resolution sensors while working near the bottom. In order to maximize the spatial range of the sensors, GREX set up a team of vehicles that moves in formation and thereby broadens the operational swath width of the system, demanding synchronized motion control, decentralized decision-making, and inter-vehicle coordination. However, owing to the lack of advanced autonomy of the vehicles employed in the project, the pre-planned navigation configuration was restricted to more or less flat regions, although geologically or biologically significant areas typically present a wide variety of relief. The prevailing operational methodology to address this problem is the participation of human operators in the loop utilizing tethered vehicles.

The European project MORPH proposes a significant move forward on this issue (Kalwa et al., 2016). The concept is that a group of heterogeneous cooperative vehicles or self-propelled sensors carry out a multimodal survey of underwater structures. Spatial disparity provides the multiple points of view needed for both high-resolution surveys and the detection and prevention of obstacles. The separation between nodes enables various sensing systems to work according to their specific optimum range, e.g., Sonar nodes are farther away from targets than camera nodes. The diversity of vehicles even allows for a better combination of navigation and localization data, e.g., nodes adequately separated from interference structures may provide an external navigation guide to nodes near to these structures. More specifically, this decentralized and physically separated configuration of the system allows for morphing, i.e., the fleet will rapidly respond in real-time to perceived variations in the real world that can not be accounted for by *a priori*. MORPH explores and tests a range of interdisciplinary problems relating to fleet navigation and control, secure and efficient morphing, feedback and information affecting morphing, fleet design and knowledge sharing, C2S for useful mission management.

A successful interoperable C2S initiative was initiated in 2005 (Dias et al., 2005) by the Porto University—Underwater Systems and Technology Laboratory (LSTS). The NEPTUS architecture goal is to enable integrated operations of heterogeneous UMVs teams, operating sea, ground and air vehicles and individuals. People also play a key role in autonomous vehicles, where a mixed-initiative process is necessary. The operating situations for these teams are primarily environmental protection operations, but they may also include environmental disasters, rescue missions, etc. The distributed architecture of Neptus is a service-oriented architecture that enables high degrees of interoperability (between applications), scalability (number of nodes), and reconfiguration (number and kind of nodes). NEPTUS has been used in many at-sea tests such as the Rapid Environmental Picture Atlantic exercise 2014 (de Sousa et al., 2015). This experiment, involving more than 10 military and civil organizations, emphasized multi-domain missions in order to foster interoperability and cooperation between UMVs and aerial vehicles. The NEPTUS toolchain supplied an unified C2S that allowed the integration of numerous vehicle systems, enabling wireless and underwater interoperable communications and destructive delay-tolerant networking (DTN) capabilities.

More recently, the Widely scalable Mobile Underwater Sonar Technology (WiMUST) H2020 project (Abreu et al., 2016; Indiveri et al., 2016) validates at-sea a system of cooperative UMVs for geotechnical measurement and geophysical mapping. The new core innovation of the WiMUST framework is the use of a team of collaborative autonomous underwater robots, functioning as intelligent sensing and communication reconfigurable mobile acoustic network. The project brings together a community of academic organizations, geophysical survey firms and SMEs with an established track record in autonomous adaptive technologies, networked cooperative control and navigation, and marine robot architecture and manufacturing. Bottom surveys are nowadays collected from side-scan or multi-beam sonars, which are towed from ships or embarked on autonomous vehicles. The WiMUST concept offered a technological breakthrough in the development of robotic distributed sonar system with autonomous mobile nodes, making operation at sea much easier given the fact that no physical connection exists between the surface ship and the acquisition equipment. The final demonstration in the Atlantic Ocean involved ten heterogeneous vehicles but all the technologies designed during the project were conceived and implemented with a specific long term vision: underwater missions performed by a large number of autonomous cooperating robots.

Moreover, the system presented in Robb et al. (2018) dealt with the difficulty of monitoring multi-objective, multi-vehicle operations, while at the same time resolving the ambiguity about the actual status and the protection of distant, high-value platforms. In order to increase the reliability and efficacy of UMV C2S, the authors suggested a hybrid of an interactive, natural language operator interface coupled with communications using multi-domain channels to transmit data through various devices and delivery modes, enhancing C2 of coordinated and ultimately autonomous missions. The Multimodal Intelligent inteRactIon for Autonomous systeMs (MIRIAM) natural language interface enables operators to straightforwardly update an autonomous network about the progress of the mission goals and the state of the AUVs assigned to it (Hastie et al., 2017). MIRIAM has the capacity of connecting to commercial C2 and applications, collects continuously updated task and vehicle data, acknowledges user requests, supply outputs, and produces its own messages of significant issues in natural language. They have successfully demonstrated their interoperable systems at sea using the OceanServer IVER-3 AUV as the vehicle to be operated and tracked, the EvoLogics Sonobot USV as the communication gateway and the Seebyte Seetrack-Neptune C2 program with the MIRIAM natural language interface in the on-shore C2S.

Last but not least, all the models, hardware and software composing a system-of-systems must pass a *VV&A process* (Hodicky, 2014). The article underline how the analysis of potential integrations of an Autonomous System (AS) into the operational field must be a priori tested to keep costs low, i.e., through M&S systems for experimentation in synthetic distributed environments. These environments are based on cooperating entities using data interchange mechanisms such as the High Level Architecture (HLA) (Möller et al., 2008). It is a suitable candidate due to its maturity and broad adoption (e.g., it is the only distributed simulation framework accepted as a NATO standard—STANAG 4603). Its most recent and major improvement is the Federation Object Model (FOM), which acts as a shared vocabulary for communication between M&S systems. The creation of an AS common vocabulary in M&S can even increase the potential of the synthetic experimental framework to ensure the easiest and most effective way to implement AS in the operational field. Although there are also tight industry standards for networked system, there are no exhaustive methods to lead the researchers through the VV&A cycle for the evolution of autonomous interoperable systems. In order to ensure coherent outcomes in all network simulation configuration, new practices are necessary because of the nature of the system-of-systems relationships in a decentralized environment which simulates exchanges between autonomous assets (Hodicky, 2018). For VV&A of modular network of systems, (Tremori et al., 2018) introduces an agile M&S architecture which enables better knowledge of the whole system by testing together all its component (i.e., hardware and software) in virtual-reality environments that are operationally meaningful. The functional architecture suggested adheres to the most recent Institute of Electrical and Electronic Engineers (IEEE) recommended practice for VV&A (IEEE, 2007) and NATO guidelines in the sector (Ruiz et al., 2016).

Finally, it is worth to mention that the CMRE started a multi-year project in 2014 entitled Persistent Autonomous Reconfigurable Capability (PARC) aimed at assisting NATO in preparing the future in this domain and addressing common technological shortfalls, cost aspects and challenges related to the transition of this type of technology. The objectives of PARC include increasing maritime unmanned systems' persistence, *interoperability*, scalability while addressing standardization, information assurance, and cost aspects. Two examples of remarkable results can be found in Carrera et al. (2016) and Petroccia et al. (2018). In the first, an HLA connection between simulated assets and an autonomous system using the Robot Operating System (ROS) middleware is provided, enabling both M&S and robotics researchers to develop more complicated and accurate simulations of operationally relevant environments. The latter reference is a description of the AutoLARS (Launch And Recovery System) system which allows AUV docking, wireless battery charging and high data-rate download of collected data with the ultimate aim of improving the persistence of these systems far beyond their batteries limits.

Besides PARC, CMRE is constantly involved in promoting *Standardization Agreement (STANAG) also about interoperability*. Among NATO members, a STANAG establishes methods, requirements and constraints for operations and systems. The aim is to provide joint procedures and logistics so that the military of one Member State can interoperate easily with the others. STANAGs are also the foundation for interoperability between a different Information and Communication Technology (ICT) systems, which is crucial for NATO and allied missions. Two of them are very important and worth mentioning in this paper: 4586 and 4748.

First, the STANAG 4586 (Marques, 2012) is the current standard for Unmanned Aerial Vehicles (UAV) (Platts et al., 2007; Frazzetta and Pacino, 2013) and it is in place a study group on Multi-Domain Control Station (MDCS) which aims at going toward a “joint” standard, i.e., one that will cover air, land and maritime unmanned systems. The MDCS working group “a mixed industrial and government representatives group” will likely turn its ideas into a STANAG (i.e., an updated version of the STANAG 4586) in the future. Secondly, CMRE, together with academia and industry, created an underwater communications protocol known as JANUS (Potter et al., 2014), recently advertised as NATO STANAG 4748 (NSO, 2017). It is the first globally accepted and openly accessible communications standard for all the communities of the underwater domain (http://www.januswiki.com). It is built on the binary frequency change with tunable center frequency and bandwidth. The subsequent bit rate is 80 bps if the default channel is selected (9.4−−13.6 kHz band). Automatic Identification System (AIS), meteorological and oceanografical data transfer to submarines, other than assistance in distressed submarine operations, are distinctive functions of JANUS (Alves et al., 2016; Petroccia et al., 2016, 2017). Thanks to the use of a standardized approach, it will be possible to raise the level of Maritime Situational Awareness (MSA) as well as to enhance security and water-space governance employing heterogeneous and hybrid systems, both manned and unmanned, including collaborative UMVs networks. In conclusion, JANUS is the first comprehensive solution that makes it possible to standardize communication protocols at the physical level between multi-vendor devices, acting as a fundamental *glue* between the existing proprietary protocols.

Another very active player in the interoperability field is the European Defense Agency (EDA) since its 2008 Unmanned Maritime Systems (UMS) programme (EDA, 2011; Dahlmann et al., 2015), which established a list of key-technologies needed for the appropriate functioning of the UMVs irrespective of naval application. The whole programme aims at coordinating efforts from individual member states to foster interoperability, safety and more broadly the use of UMS. A specific UMS-project has been launched (called STANDIN: Standards and Interfaces for more interoperable European UMS) to take into account information on standards/interfaces from UMS-projects. The STANDIN project aim is to provide a relevant recommendation, identifying issues that may hinder the eventual achievement of the UMS-programme objectives. The endorsement of the recommendation of the STANDIN project should enhance innovation (use of common interfaces/standards to enable industries to produce components to be easily integrated and tested on UMS), upgradability of UMS and plug & play. However, the recommendation is not expected to be translated into new regulated standards but instead it will depend on the will of governments and industry to enforce the recommendation, i.e., adopting it as a “de-facto standard” for European UMS.

The last but fundamental element of interoperability is the adoption of a specific *robotics middleware*. These intermediate software level is the fulcrum of interoperability between UMVs. A robotics middleware can essentially be thought as a software layer, composed of several modular packages, which has the task of collecting and harmonizing the information coming from the on-board sensors, making them available to the various processing nodes. These nodes must in turn process these data according to their specific function (tracking, communications, etc.) and pass the outputs to other nodes or directly to sensors and/or actuators of the robotic system. At the end of the various elaborations, the middleware will be the responsible for the passage of the system in another state, e.g., the execution of a specific action.

The current *de facto* standard middlewares for UMVs include MOOS [11] and ROS [12]. They are both publish-and-subscribe systems, which provide the communication of arbitrary data throughout a network. However, in order to complete mission objectives, a robotic system also requires a deliberative component in addition to its reactive aspects (e.g., avoiding obstacles) (DeMarco et al., 2011). This is the goal of the MOOS process IvP-Helm, which act as an autonomous decision-making engine that executes in the backseat of the robotic platform (Benjamin et al., 2010). In fact, the MOOS-IvP is a combination of two components, IvP Helm and MOOS. While MOOS is the actual robotics middleware, abstracting a TCP/IP based inter-process communication (IPC) protocol, IvP is a multi-objective optimization framework for constructing complex autonomous behaviors from a collection of more simple routines. The whole environment is composed of several other processes, which communicate using the MOOSDB database as a broker. The rate of interactions across information channels, called topics, is controlled tightly by the MOOSDB, occurring synchronously at a predefined rate. The exchange of information occurs between TCP ports, which may or may not exist on the same physical machine. Therefore, MOOSDB is relatively rigid, and each topic is bound to a startup-defined data type, with induced additive latencies because of the travel path of information (node-database-node).

On the other hand, ROS does not impose any architectural constraints, i.e., processes (called nodes) communicate directly with each other, with no central broker. A central node (called master) exists, but only to manage node startup and shutdown. This is the main differences between MOOS and ROS: while all data within a MOOS system are transmitted through the MOOSDB, in ROS data are transferred using peer-to-peer communications. The basic unit of interaction in ROS is a message, which is typically exchanged on a topic: from a node's perspective, messages are published synchronously and read from a subscription asynchronously. Furthermore, a single topic can contain multiple instances of a message. In ROS, there are two extensions to the typical publish/subscribe mechanism: *services* and *actions*. A service simply takes an input and returns an output, as a typical service does in computer science literature. An action keeps an internal state on a longer time scale because it is called with a goal, emits feedback during the action, and finally returns a single result at the end of the action. An important advantage of ROS over MOOS is its capability of handling very large datasets, e.g., live high-resolution video and multi-dimensional point clouds from LIDAR-like devices.

The development of MOOS-IvP in the research community is continuous and ongoing, while its usage in the industry is very limited. This may be a consequence of the lack of insurance on backward compatibility that is based on community agreement, not on enforced standard (even if newer versions are generally backward compatible). While MOOS has historically been popular within the underwater robotics community, ROS is now by far more pervasive in a multi-domain context (ground, sea, air). The main reasons for such success are the reconfigurability and ease of use of ROS. There are bindings for both C++ and Python, and it is regularly updated (at least yearly). There are no rigid standards guiding development, but it is so widely used that there is extreme prejudice against breaking backward compatibility.

Another popular middleware in the marine robotics community is DUNE (DUNE: Uniform Navigation Environment) mainly due to its usage in autonomous vehicles designed by the LSTS Group at the University of Porto (Pinto et al., 2013). DUNE offers a C++ programming framework for robust and flexible real-time reactive operations, and also uses the publish/subscribe method. DUNE modules, named tasks, publish and subscribe messages without requiring any specific knowledge of the other tasks. For most instances, basic interface modifications are sufficient to implement new features. Communication between tasks is carried out solely through a message-oriented Inter-Module Communication (IMC) protocol for UMVs and sensor networks (Martins et al., 2009).

Through an industrial point of view, the Common Object Request Broker Architecture (CORBA) is worth considering as a middleware standard (https://www.corba.org/). It was launched in 1991, supported by the Object Management Group (OMG) and commonly adopted by major organizations such as Thales, Raytheon, and BAE. The current edition dates back to the end of 2012. Several implementations are accessible, such as omniORB, PrismTech, and RT-CORBA, which share the primary strength of CORBA: framework and applications are separate, enabling vendors to interoperate based on the interface description language (IDL) specification (Henning, 2008). The successor to CORBA is the Data Distribution Service (DDS), an IPC standard specification initiated in 2004 by the OMG standardization committee (a description of the DDS standards can be found at https://www.omg.org/spec/category/data-distribution-service/). There are several industrial implementations relevant to companies involved in autonomous vehicles or ground control networks. The DDS is a language-agnostic IPC standard, and it does not recommend a specific implementation of any sort neither a device communication framework. DDS is a standard that is preserved and strengthened by OMG, an agency that carries a great deal of weight in the industry. However, as a successor to CORBA, the development of industrial migration to the newer standard is not obvious. Additionally, although the specification is fully available, most of the implementations are private, with no outstanding candidates in the open-licensed domain.

Finally, the Joint Architecture for Unmanned Systems (JAUS) is worth noting as a concrete middleware standardization initiative. JAUS is a specification directed at unmanned systems, introduced by the US DoD to provide a basis for interoperability between unmanned systems (Whitsitt and Sprinkle, 2011). In order to guarantee that the device design is valid to the whole domain of existing and future unmanned systems, the JAUS Reference Architecture (RA) was focused on five principles: vehicle platform independence, task autonomy, computer hardware independence, technology independence and operator independence. This design has passed from the JAUS Working group, which consisted of individuals from government, industry and academics, to the Society of Automotive Engineers (SAE). The Technical Committee of the SAE Unmanned Systems now retains and promotes the collection of standards. Most specifications have been transferred from the JAUS Reference Architecture to a services-based system including for example AS5669 (JAUS Transport Standard) and AS5710 (JAUS Core Service Set). In particular, the AS5669 (https://www.sae.org/standards/content/as5669a/) specifies the transport layer between processes (header compression, source/destination address, TCP, UDP, or serial link) but does not identify lower-layer (data link-physical) operations and is therefore independent of the medium from which the messages are sent. This characteristic improves interoperability between distributed networks and inter-node (or vehicle) communications. Before the “official” SAE JAUS version, the JAUS RA was developed to provide first-time developers with an open implementation that would be comparable to the norm. However, when the first pay-for edition of SAE JAUS was released, there was considerably less open-source support. Several of the initial free implementations compliant with JAUS RA have been scrapped, and others have vanished behind new proprietary licenses and other pay-as-you-go systems. To sum up, the JAUS standard claims to be well-written, but it has not been broadly implemented in the underwater scientific field because the standard and several of its applications are proprietary, raising obstacles to entry and reducing visibility for research groups.

## 3. At-Sea Experience: The ASW Operational Deployment of Concepts '17

This section briefly summarizes the work presented in Costanzi et al. (2018): besides describing how the *interoperability issue* was approached during an experimental campaign held in October 2017, it provides more information on the overall network and the deployed components to emphasize the complexity of the scenario and to underline the high level of interoperability reached.

**Figure 1:**
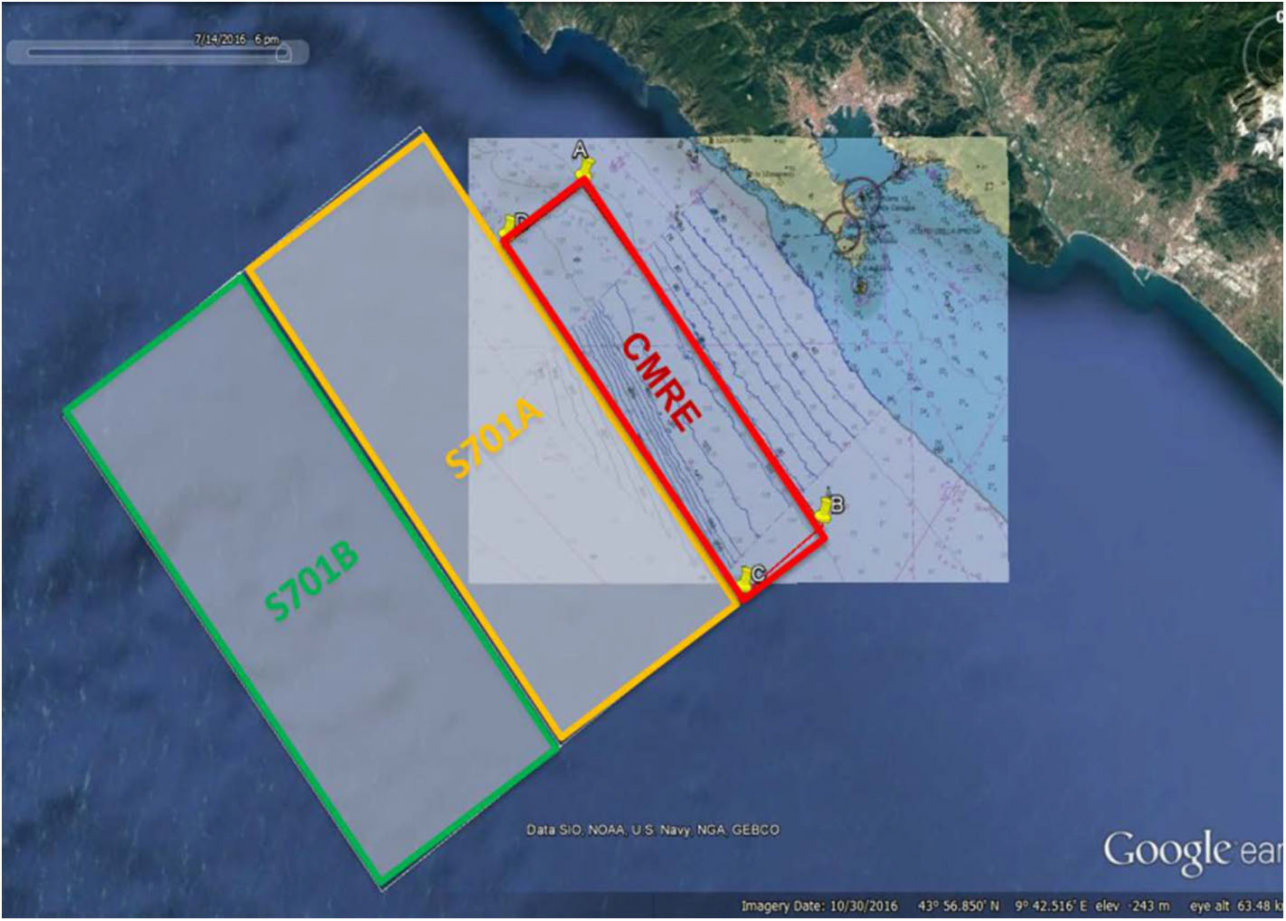
Map of the area off Cinque Terre, La Spezia, Italy, requested for ASW-ODC17 sea trials (snapshot from Google Earth) in the period 12–17 OCT 2017.

The ASW-ODC17 experimentation aims, among other objectives, to demonstrate interoperability of different legacy and modern systems, like the Folaga WAVE glider-AUV, within the CMRE network. This kind of operational experimentation is vital to receive UMVs requirements from the end-users and to demonstrate to them the potential capabilities and challenges of integrating unmanned systems with legacy maritime assets. Although CMRE has always worked with other naval units on the margins of other operational exercises on a non-interfering basis, this is the first time that it has been allotted a dedicated period to conduct trials with the NATO standing forces. The ASW-ODC17 experimentation area was defined by the three boxes shown in Figure 1. Almost 20 nodes were involved during the experimentation, including static and mobile assets, both manned and unmanned. The remarkable extension of this network poses an actual interoperability challenge.

The backbone of the experimentation was the Network Enabled Modem Operator (NEMO): the CMRE software framework designed with MOOS-IvP to support experiments in mixed media (air and underwater) (Vermeij et al., 2015) using underwater communications in data exchanges between mobile and potentially autonomous nodes (unmanned to unmanned) and C2. NEMO represents a move in the direction of a more versatile marine communication infrastructure which will leave behind pAcommsHandler's heritage, that was the first IvP process operating the vehicle's acoustic modem (Freitag et al., 2005). NEMO provides a workspace where efforts such as JANUS, SDOAM, clock synchronization or routing can be deployed, tested and implemented. The NEMO is the current marine communication stack for CMRE research, which still prefers MOOS-IvP mainly because of the IvP part. It allows the vehicle to work autonomously toward a goal, while it operates within mission requirements, operating an efficient de-conflicting of the tasks. Anyway, due to their many similarities, there is almost a one-to-one correspondence between ROS and MOOS system calls, and the path forward for the Centre is the integration of the two middlewares. The next step will be the development of a cognitive communications architecture (CCA) that allows other channel access outside TDMA (Time Division Multiple Access), utilizing intelligent, adaptive and secure submarine networking strategies (Petroccia et al., 2018).

**Figure 2:**
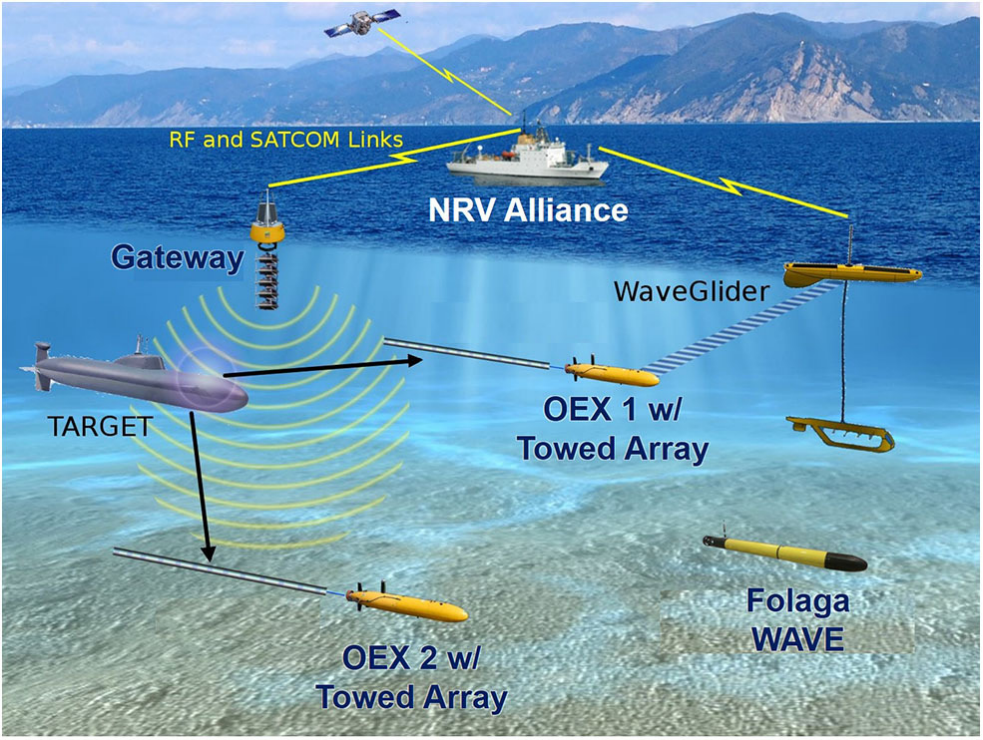
A descriptive outline of the multistatic network for ASW of the CMRE with the integration of the Folaga-WAVE. In this ASW architecture, multiple active sonars are located on ships and buoys, and cooperate with multiple receivers, i.e., towed arrays installed on OEX AUVs and other manned assets. The communications between all the platforms, underwater and above the water surface, are ensured by the WaveGliders, acting as mobile gateways, together with the moored gateway buoys. The CMRE OEX AUVs, called Groucho and Harpo, are the mobile sonar receivers. The NRV Alliance is the network C2 centre and is also part of the network, towing both a source and an array. For the specific experimentation, the Folaga WAVE was equipped with a CTD probe, the data of which were made available for periodic updates of the environmental map in the area and for insertion into the acoustic engine, in which the onboard processed sound speed can be a valuable information.

The physical cores of the CMRE's multistatic hybrid network for ASW were the active sonars (i.e., acoustic transmitters installed on a buoy and towed by NATO Research Vessel Alliance) and a typical scenario can be seen in Figure 2. In particular, the new triplet SLIm Cardioid Towed Array (SLICTA) array (Canepa et al., 2017) was towed from NRV Alliance along with the ATLAS Source and receiver array. When the transmitted pings are scattered by objects, receiving hydrophone arrays can collect those echoes from different positions, including the arrays towed by autonomous platforms (in particular two Ocean Explorer—OEX—AUVs). The two OEX AUVs owned by CMRE have been fitted with acoustic modems and they can tow an array developed and fitted for purpose, i.e., the SLICTA. CMRE has successfully tested—during ASW-ODC17—a paradigm for the allocation of robotic assets to ASW tasks, a problem called the Multi Robot Task Allocation (MRTA) (Ferri et al., 2017). In the underwater field, centralized network monitoring, which can enable tasks distribution optimization, is not feasible for the outlined problems and peculiarities of the channel, and this is why interoperability is so crucial. The suggested scheme of assignment operates in a fully federated manner and two simultaneous auctioning nodes handle the actual tasks. The CMRE MRTA utilizes adjacent node agreement and only needs local exchange of underwater data.

Also WaveGliders, UMVs that use wave motion to navigate (Willcox et al., 2009), were employed in the experimentation along with a set of deployed moored buoys to create a communications network that allows feedback, localization and exchange of control and information between manned and unmanned platforms (Munafò and Ferri, 2017). The Alliance has the role of C2S to allow the users to communicate in real-time with the network through multi-hop communications, via undersea or RF connections. This allows the Alliance to remain far from the patrolled area with the possibility to undertake additional operations. The main benefit of using various assets, active and passive, is the expansion of the network range employing the specific multistatic geometry to augment the probability of sonar identification with sonar signal processing.

During the experiment, the Italian Navy agreed to supply the Leonardo Coastal Research Vessel (CRV) equipped with a towed echo repeater to emulate an acoustic scatterer by retransmitting the sonar signal recorded from the source according to user-specified parameters (e.g., delay, attenuation, etc.) (Grimmett, 2009).

**Figure 3:**
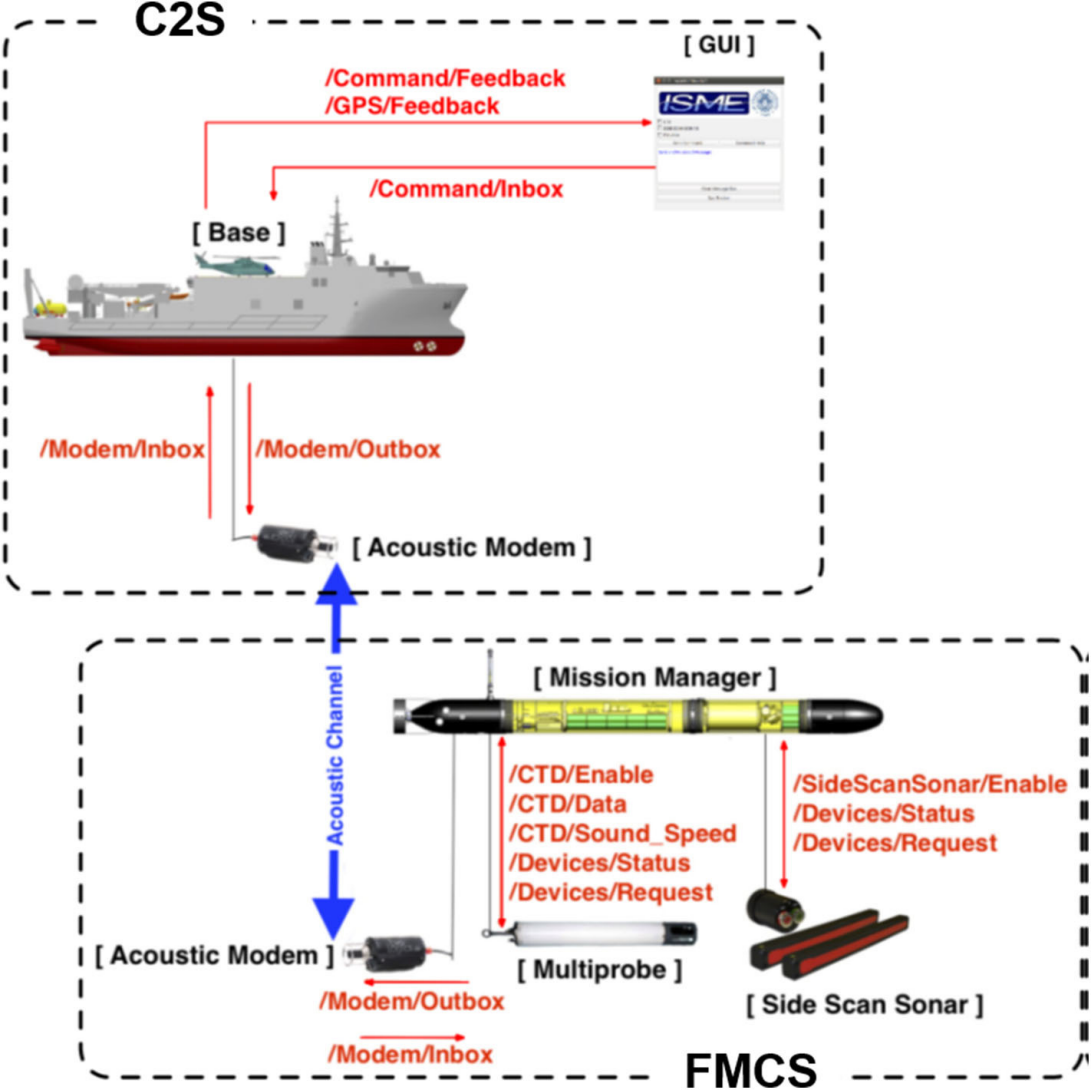
Conceptual scheme of the WAVE Mission Control System (WMCS). The modularity of the WMCS allows both to displace nodes on different platforms in different domains (above, on, and under the surface) and to easily add new mission payloads simply by maintaining the interface and message architecture defined at system level. In fact, the [Acoustic Modem] and part of the [Base] ROS modules have been installed on the gateway buoy during the experimentation along with the ROS-MOOS bridge. Figure from Costanzi et al. (2018).

Finally, the WAVE vehicle—capable of navigating using wave motion and recharging with solar energy (Caiti et al., 2018)—was added to the network to stress interoperability and add significant data to the ASW network. The WAVE Mission Control System (WMCS) combines the modules specifically developed for the project with those already existing in the AUV and ensures a high level of abstraction for the user set-up of an autonomous mission (the conceptual scheme of the WMCS is illustrated in Figure 3). The term “high level” refers to the user not having direct control of the hardware (sensors and actuators) that are installed on the vehicle but interacting with them via the implemented WMCS request-response ROS-based mechanism.

In order to be effective, efficient and reliable, the WMCS has been designed and implemented foregrounding requirements such as modularity, scalability, reconfigurability, user-friendliness, and robustness. These requirements were set in the WAVE project to meet future interoperability need, as shown in section 4. The WMCS consists mainly of two subsystems:

- The Folaga Mission Control System (FMCS) onboard the vehicle, responsible for the management of mission payloads and interaction with the low-level control system of the Folaga vehicle;
- The WAVE C2S on the base station (positioned ashore or on a support vessel), which provides essentially a mission-managing level and a graphical user interface with all the tools to carry out mission supervision and control.

**Figure 4:**
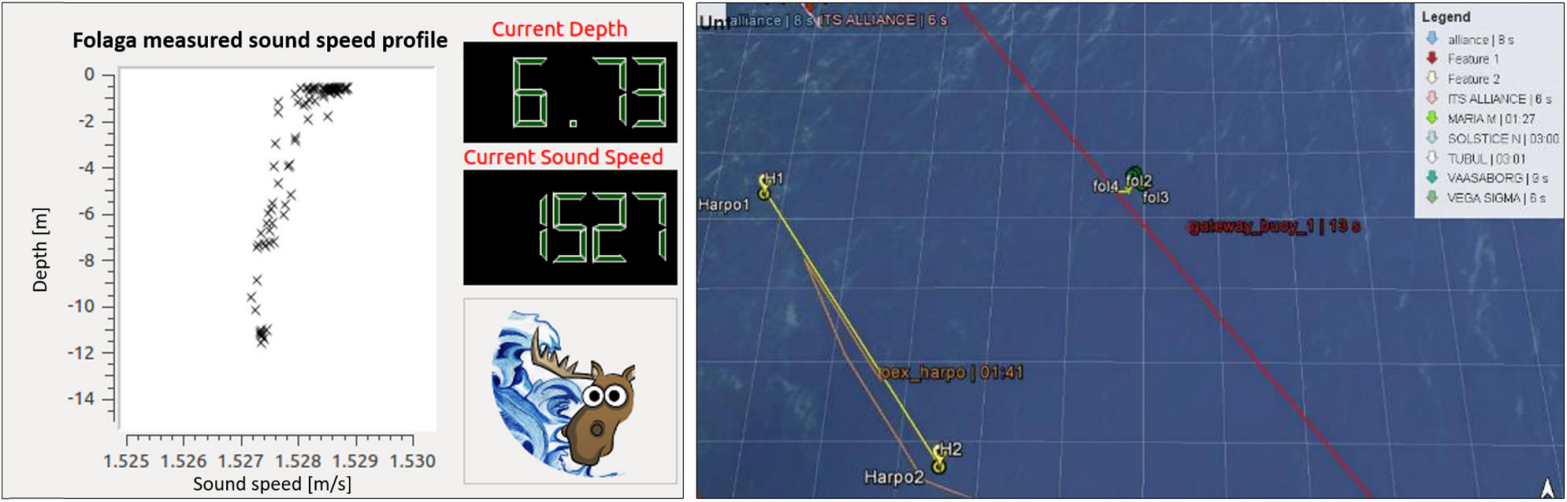
Screenshots of real data during the experiments. On the left, the sound speed received acoustically in real time during the Folaga WAVE profiling. On the right, the CMRE C2S with the AUVs' positions displayed. Figure from Costanzi et al. (2018).

Then, the WMCS implemented a distributed architecture: software modules were split between the WAVE C2S, the moored gateway buoy and the vehicle itself. While these systems have a physical separation, they merge acoustic communications (modems were installed on both the gateway buoy and the vehicle) with RF communications (between C2S and the gateway buoy, but in case also between C2S and the vehicle). This allows the user to send commands to the vehicle and receive required notifications and data. Indeed, the interoperability of the WAVE vehicle into the aforementioned CMRE heterogeneous network was demonstrated by exchanging both commands and data. In particular, the WAVE vehicle was equipped with a Conductivity, Temperature, Depth (CTD) sensor to obtain representative operational information that was communicated to CMRE Environmental Knowledge and Operational Effectiveness (EKOE) team (Grasso et al., 2016). Furthermore, CTD data has been distributed for periodic updates of the environmental map in the area and for injection into the MultiStatic Tactical Planning Aid (MSTPA) decision support tool (Strode et al., 2012), to which the sound speed measured by the vehicle can be relevant. Interaction with the EKOE team in particular enabled the view of the positions of all the underwater assets (CMRE OEX AUVs, WAVE, and CMRE Wavegliders) at the EKOE C2S. In addition, the position of the WAVE vehicle was made readily accessible on board the Flag-Ship of the NATO partners and at the command and control stations of NATO Allied Maritime Command (MARCOM). Figure 4 shows the information as they were seen on board NRV Alliance.

As it is now clear, one of the main challenge lays in the different robotics middleware of the Folaga WAVE vehicle and the CMRE network which uses ROS and MOOS respectively. Therefore, a ROS-MOOS bridge software was installed on a moored buoy acting as a gateway between underwater and surface assets. Due to the characteristics of the different AUVs participating in the experimentation in addition to the WAVE vehicle, the gateway was fitted with acoustic modems working on different frequencies. This way, all the interoperability tests rely on a double channel communication: acoustic between the gateway buoy and the vehicle, RF between the Alliance C2S and the gateway buoy.

**Figure 5:**
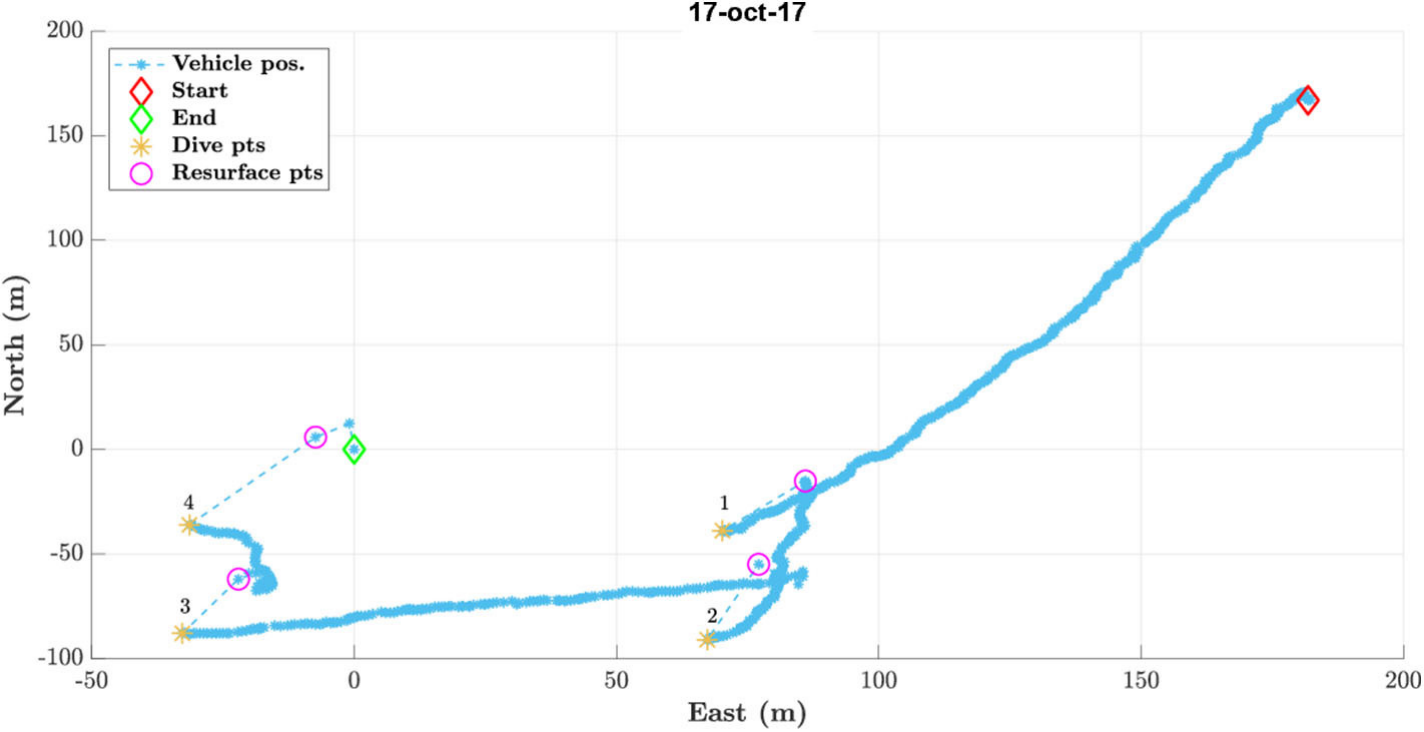
The performed interoperability test mission in NED coordinates. The vehicle was moving in gliding mode for about 250 m in the first path between the starting point (red diamond) and the first dive point (orange asterisk). Then, it started doing four profiling tasks to characterize the water column down to 12 m depth on a rectangular area of 5, 000 square meters. Recall that the vehicle simply dives vertically using the ballast and the internal moving mass for profiling the water column. Only the vertical jet-pumps are used for finely trimming the pitch angle to 0 degrees, i.e., it is free to drift during these kinds of missions. As it can be seen, it was present a strong sea current toward North-East so that the vehicle resurfaced about 30−−40 m away from each diving points. Finally, the first path from the red-diamond to the dive point no. 1 was done in surface navigation in this specific plot. Figure from Costanzi et al. (2018).

The complete mission done for all the considered experiments is shown in Figure 5. It is important to recall that all the tasks implemented on the AUV for the specific mission could be added, started, halted or terminated via acoustic modem or Wi-Fi. The integration of WAVE Folaga within the CMRE network, aimed at enhancing the interoperability in a multi-vehicle operation, was the best demonstration of the effectiveness of the system developed in the project in an operational context.

## 4. Future trends of Interoperability

System modularity and interoperability (between heterogeneous systems) are two keywords of the current world of marine robotics as an unprecedented growth of sensors, communication architectures and protocols, manned and unmanned platforms, and software at various levels is ongoing. One of the main reasons for their relevance in modern engineering developments is that modularity and interoperability ensure the reduction of the costs of a system—albeit large—along its entire life cycle, both from a maintenance point of view and from the possibility of adaptation and evolution based on the growing applications and new technologies available in the future.

Today, several factors limit the interoperability of systems, not just UMVs. Among them, the main ones are certainly the proliferation of proprietary interfaces, often followed by a non-standard physical communication architecture characterized by non-shared waveforms, frequencies and settings. Besides, much of the engineering effort is now focused on standardizing the type of data, metadata and related encoding exchanged between systems.

The development of specific main techniques and technologies can tackle these changing requirements while mitigating the effect on the platform itself:

- *Multiformat aggregated data processing:* the capacity to concurrently communicate with, manage and elaborate different data formats. If it were possible to know beforehand the data type and information formatting exchanged (including metadata and labels), as well as being able to implement processing algorithms capable of handling different data formats agnostically, platforms can be then more synergistically updated in relation with emerging operational needs.
- *Federated Distributed computing:* the capacity to quickly change modules (“plug and play” manner), while barely physical requirements have to be addressed. Once a new payload has been physically installed in compliance with the requirements of modularity and interoperability, it is necessary to test, analyze and certify the behavior of the whole autonomous system with a high impact in terms of costs and development times. Federating the computing capabilities of the system up to the payload level or even to the remote control station could be an important lever to facilitate the rapid and effective integration of new technologies.
- *Open Standards, Architectures and Equipment will improve Interoperability:* the vision currently shared in the industrial world of UMVs is that only by developing new platforms, systems, payloads, software according to common standards make it possible to allow full interoperability of the various unmanned autonomous systems. At the same time, this will enable the interchangeability of the various modules of a platform respecting today's budget constraints. Once standards have been established, the massive use of Commercial-Off-The-Shelf (COTS) components will allow the sharing of various functional subsystems between different vehicles, such as payloads, navigation systems, power supply systems, communication systems, sensors, and launch and recovery systems. Hand in hand with the hardware, the software must also comply with a standard architecture that can facilitate changes to the configuration of a system when replacements or additions of entire vehicles or specific payloads are needed. This sharing of standards at each level between heterogeneous vehicles will have positive effects from the platform's acquisition until its disposal without loss of interoperability between systems. The larger and more expensive the UMV, the more customized and proprietary interfaces will be prohibitively expensive to develop and maintain, and therefore the greater the advantage associated with the use of open standards, architectures and equipment.
- *Collaborative, Opportunistic, Advanced Communications:* the communication required in modern applications is no longer merely a point-to-point communication. Today, we have flexible and adaptive networks working in a multi-channel environment that lays challenging constraints on the performances obtainable. In particular, one of the major limitations of the underwater acoustic channel is the low bandwidth, which requires a high level of discrimination in the exchange of information (necessity, extension, etc.), favoring cooperative strategies between UMVs to take advantage of their autonomous capabilities even in the case of data loss. Moreover, these peculiarities of the underwater acoustic channel impede the use of classic collaboration algorithms used in terrestrial and air domains based on the consensus theory (Ren et al., 2005). In fact, these algorithms require an information exchange overload for network management directly proportional to the network size. An alternative solution under maturity is the use of non-acoustic communication (mainly Light Emitting Diode systems and laser) at range below tens of meters, especially for transmitting high-speed data between close UMVs and between UMVs and node acting as gateway between the underwater and non-underwater environment (for example surface vehicles or buoys with satellite connections, Doniec et al., 2010); a remarkable example of such concept is the dual acoustic/optical modem of WHOI able to adaptively operate according to the relative distance between UMVs (Farr et al., 2010). WHOI was also a pioneer in the flexible use of the limited acoustic channel through the introduction of the Compact Control Language (CCL). CCL is a series of messages that contain UMV commands and data messages for standard sensors (Stokey et al., 2005). CCL commands include basic procedures such as “Abort Now” and “Abort to Mission Start” but also sophisticated commands such as a side-scan sonar redirection over the operational area. The open design of the specification enables vehicles produced by various academic organizations or commercial firms to work together using standard data formats. New signals can be introduced by users if required for new operations, both military and civilian. In addition to pure communication between UMVs, it is necessary to determine the position of nodes within a network, and this can be done with the same autonomous cooperative approach between UMVs: simulative and experimental results have been presented for example in Allotta et al. (2014), Ridolfi et al. (2018), and Masmitja et al. (2018). These recent works focused on underwater Multi-Target Tracking (MTT), evaluating the potential to use surface vehicles as mobile markers to locate and map a set of underwater vehicles. This collaborative solution minimizes the main downside to multi-target cooperation, which is the uncertainty in underwater positioning due to the environmental uncertainty. In the mentioned studies, various network levels of sensors, nodes, and vehicles operate together, opening new possibilities for detecting and understanding the complex dynamics of ocean phenomena and creating new applications. In the civil sector, for example, there is an immediate and worldwide need for a technology that can allow environmental response teams to quickly identify the nature and magnitude of unintended leaks of toxic products to have an appropriate response. A cooperative robotic system, consisting of two heterogeneous UMVs, for environmental control is introduced in Vasilijević et al. (2017). The described hybrid surface-underwater architecture enables the operator to interpret the product concentration data in real-time, using the system's modeling and decision-making abilities, and to adjust the task on the move. The system is an application of the Human-on-the-loop (HOTL) concept (Cummings et al., 2012), which allows a minimal team of operators to manage a network of robotic agents working in complex, time-consuming environments. The tests demonstrated enhanced process efficiency for a network of autonomous vehicles in search, track, and neutralization missions. The HOTL principle is compared vs. the human-in-the-loop principle in Valavanis and Vachtsevanos (2015), highlighting the unique technological challenges and degrees of autonomy needed by autonomous vehicles to carry out a task without substantial human involvement. HOTL supports decision-making and helps the operator to conduct the most suitable task in an evolving scenario, i.e., to enable on-the-fly mission adjustment.
- *Interoperable, realistic integrated Modeling and Simulation environment:* approaches consisting purely of in-field experimentation in the marine robotics domain are prohibitively expensive; thus, the interoperability of UMVs and heterogeneous integrated platforms and systems will probably be critically assessed using modeling and simulation. The future trend of M&S may be focused on the design of a scalable architecture for interoperable simulation based on a system of systems approach, providing a V&V (Verification and Validation) capability to explore systems reliability in complex conditions and to analyze autonomous behaviors in cost effective and safe virtual environments (Hodicky, 2016). The paper sets out the importance of exploiting Augmented Reality and haptic feedback to deliver immersive simulation in a natural way to the human operator who is working with autonomous systems. Such simulations would make it possible to address the human factor, i.e., to create a condition similar to that faced in real operations. The modeling of human factors, such as stress, will therefore represent a future challenge for M&S systems. To sustain the UMVs development, M&S systems must be designed according to the IEEE Distributed Simulation Engineering and Execution Process (SISO, 2010), and the more recent Scenario Development Guideline of the Simulation Interoperability Standard Organization (SISO, 2016). Future M&S will consists of a network of simulators working together with C2 stations in the loop and with special emphasis on implementing autonomous behavior of UMVs and their messages transfer using standard procedures, e.g., the C2-Simulation Interoperability (C2SimI) language (Tolk and Boulet, 2007). In Biagini et al. (2018), the authors describe an M&S federation of simulators communicating with operational C2S. The simulator federation is based on the HLA Run-Time Infrastructure (RTI) working with several technologies such as *ad-hoc* Artificial Intelligence (AI) modules for robotic behavior and C2SimI for interaction between simulators and C2S. The paper illustrates how M&S will help evaluate potential scenarios concerning an Autonomous System of Systems, promoting the development cycle not just for new platforms, but also for performance assessment methodologies and operative procedures.

## 5. Conclusions

In this paper, a comprehensive view of interoperability among UMVs is provided. Interoperability is a fundamental feature for the success of UMVs missions and its development is a long-term goal for both civil and military communities. The current plethora of UMVs is characterized by poor interoperability among them and with external systems, including legacy ones, essentially because of the urgent needs in operational theaters—for the military world—and the parallel growth of the UMV market—for the industrial and civil world. However, interoperability remains the key to increase the capacity of an operational system of systems to share information quickly, improving the MSA and therefore the efficiency in using the available resources.

The critical analysis in section 2 of the various studies and projects focusing on the concept of interoperability among UMVs, not neglecting the role of human operators in the loop, demonstrates that interoperability is currently receiving a high level of attention with a large amount and diversity of efforts. This can be explained by the fact that the current missions of the UMVs are finding increasingly blurred lines of operational space while the requirement to standardize and re-utilize sensors, algorithms, information, systems and vehicles, is urgent but still very difficult. From the analysis of state of the art presented, it is noted that currently there are no comprehensive standards on interoperability, but the groundwork effort is underway (e.g., STANAG 4586). The real challenge, following the promulgation of such a standard, will be to convince UMVs and sensors suppliers to fully adopt it due to proprietary interests.

Moving to the field, interoperability tests between the innovative Folaga WAVE AUV, equipped with oceanographic sensors, and the CMRE network C2S onboard NRV Alliance, using a gateway buoy as a ROS-MOOS bridge, are presented in section 3. Their integration has been evaluated and validated through at-sea operational experiments off the La Spezia coast (Italy). This successful interoperability experimentation between different autonomous systems, with their own acoustic modems and middleware, is an important step to improve MSA with respect to underwater assets during a joint NATO exercise. The Folaga WAVE software modularity makes it possible to incorporate a new AUV in the existing CMRE network, which in turn demonstrated its flexibility to integrate newly available assets thanks to its decentralized architecture. According to the authors' knowledge, this was the first Italian interoperable approach to be thoroughly tested and demonstrated in an operational exercise involving NATO manned and unmanned assets.

A common view arises in this paper from the theoretical and experimental evaluation of missions peculiar to UMVs: they must be fully interoperable in order to enhance their efficiency, reliability, and survivability while lowering the human burden keeping reduced cost. For future unmanned systems, modularity, interoperability and the use of advanced technology must meet more sophisticated operational requirements (section 4), while solutions developed for terrestrial networks could not effectively be extended to marine scenarios. Future challenging scenarios will require UMVs to interoperate with other manned and unmanned components of the *whole system-of-systems* to enhance the capability to gather information, make decisions, and execute actions, thus reducing reaction time. The authors' proposed challenge on interoperability is to find a transparent way to transfer control of a given UMV's payload from one “control station” to another controlling entity (human or robotic) while keeping the rest of the UMV controlled by the original one.

Finally, current operational culture poses a brake on the interoperability among UMVs. Even if out of the field, human operators are still very involved in the non-autonomous unmanned systems missions, i.e., a point-to-point communication and command line is needed and typically established. An Interoperable system-of-systems of autonomous UMVs will request the human being involved at a supervisory level only and on limited time windows. The acceptance of this concept of operations will be achieved with a progressive approach, reducing the human supervision step-by-step after establishing trust in the system's performance (in a very broad sense). The extent of human monitoring will be reduced as reliability increases, supported by stable and mature interoperability, enabling Autonomous UMVs to attain their maximum capacity.

## Author Contributions

VM wrote the manuscript with support from RC and DT. RC, VM, MS, AC, and AT conceived and developed the concept. DF, MM, and LM provided support in algorithms and C++ code for integration in ROS and MOOS middlewares. AT was the Scientist in Charge of the whole ASW-ODC17 experimentation while RC, DF, VM, MM, and LM designed and ran the specific experiments here presented. MS, AT, and AC gave feedback on the paper and helped to obtain the funding for this research.

## Conflict of Interest

The authors declare that the research was conducted in the absence of any commercial or financial relationships that could be construed as a potential conflict of interest.
